# Efforts by the National Academy for State Health Policy

**DOI:** 10.1093/geroni/igab046.674

**Published:** 2021-12-17

**Authors:** Wendy Fox-Grage

**Affiliations:** NASHP, Washington, DC, District of Columbia, United States

## Abstract

The National Academy for State Health Policy hosts both the RAISE Act Family Caregiving Resource and Dissemination Center and the Hub for State Strategies to Build and Support Palliative Care, with generous funding from The John A. Hartford Foundation. The value of supporting individuals with serious illness and complex conditions as well as their family caregivers through telehealth, care management, advance care planning, and other added family caregiver supports has been especially evident during the COVID-19 pandemic. Policymakers are now grappling with how to restructure hard-hit health care and long-term services and supports systems to better support these individuals and their family caregivers. The State Hub provides concrete resources for states working to implement and expand high-quality palliative care, and the RAISE Center is assisting the Family Caregiving Advisory Council with creating the country’s first national Family Caregiver Strategy.

